# Polyphenols from Berries of *Aronia melanocarpa* Reduce the Plasma Lipid Peroxidation Induced by Ziprasidone

**DOI:** 10.1155/2014/602390

**Published:** 2014-06-25

**Authors:** Anna Dietrich-Muszalska, Justyna Kopka, Bogdan Kontek

**Affiliations:** ^1^Department of Biological Psychiatry, The Chair of Experimental and Clinical Physiology, Medical University of Lodz, 6/8 Mazowiecka Street, 92-215 Lodz, Poland; ^2^Department of General Biochemistry, Faculty of Biology and Environmental Protection, University of Lodz, 90-236 Lodz, Poland

## Abstract

*Background*. Oxidative stress in schizophrenia may be caused partially by the treatment of patients with antipsychotics. The aim of the study was to establish the effects of polyphenol compounds derived from berries of * Aronia melanocarpa * (Aronox) on the plasma lipid peroxidation induced by ziprasidone * in vitro*. * Methods*. Lipid peroxidation was measured by the level of thiobarbituric acid reactive species (TBARS). The samples of plasma from healthy subjects were incubated with ziprasidone (40 ng/ml; 139 ng/ml; and 250 ng/ml) alone and with Aronox (5 ug/ml; 50 ug/ml). * Results*. We observed a statistically significant increase of TBARS level after incubation of plasma with ziprasidone (40 ng/ml; 139 ng/ml; and 250 ng/ml) (after 24 h incubation: *P* = 7.0 × 10^−4^, *P* = 1.6 × 10^−3^, and *P* = 2.7 × 10^−3^, resp.) and Aronox lipid peroxidation caused by ziprasidone was significantly reduced. After 24-hour incubation of plasma with ziprasidone (40 ng/ml; 139 ng/ml; and 250 ng/ml) in the presence of 50 ug/ml Aronox, the level of TBARS was significantly decreased: *P* = 6.5 × 10^−8^, *P* = 7.0 × 10^−6^, and *P* = 3.0 × 10^−5^, respectively. * Conclusion*. Aronox causes a distinct reduction of lipid peroxidation induced by ziprasidone.

## 1. Introduction

Oxidative stress plays an important role in schizophrenia and other psychiatric disorders. Schizophrenia is a chronic psychiatric disorder that affects at young age many cultures around the world. The aetiology of schizophrenia remains elusive, although several hypotheses have been suggested including abnormalities in cell membrane phospholipids metabolism or its alteration and dysfunction of cell membranes. Oxidative stress plays an important role in the pathophysiology of schizophrenia [1–9].

In schizophrenic patients, dysregulation of reactive oxygen species (ROS) generation and metabolism, as detected by abnormal activities of critical antioxidant enzymes and other indicators such as lipid peroxidation in plasma, red blood cells, blood platelets, and cerebrospinal fluid, are observed [3, 10–12]. It is well known that oxidative stress with peroxidation of polyunsaturated fatty acids (PUFA) in the membrane can lead to the abnormalities that have been observed in schizophrenia [11, 13, 14]. Oxidative stress occurs when the production of ROS exceeds the natural antioxidant defence mechanisms, causing damage to macromolecules such as DNA, proteins, and lipids. Antioxidant defence mechanisms remove the free radicals to prevent oxidative damage to biomolecules. The antioxidant system comprises of different types of functional components such as enzymatic (superoxide dismutase, catalase, glutathione peroxidase, glutathione reductase, and glutathione S-transferase) and nonenzymatic antioxidants. The nonenzymatic antioxidants include reduced glutathione, vitamin C, vitamin E (*α* tocopherol), uric acid, and various exogenous antioxidants including polyphenols. Antioxidants are substances that due to reduction of oxidative stress may prevent potentially disease-producing cell damage. The oxidative stress and oxidative injury in schizophrenia may be dependent partly on antipsychotics used and on dietary supplementation of antioxidants such as vitamins, carotenoids, polyphenol compounds, or EPUFAs that can correct membrane phospholipids. Some polyphenolic compounds extracted from berries of* Aronia melanocarpa* and used as Aronox (Agropharm/Adamed) have been reported to be protective agents in patients with breast cancer* in vitro* [15].

Lipid peroxidation is considered an important marker of oxidative stress and was found to be higher after treatment of patients with first generation antipsychotics (FGAs) [16]. Studies on rats indicate that treatment with both FGAs and second generation antipsychotics (SGAs) for <14 days does not alter the antioxidant defence enzymes and levels of lipid peroxides [17, 18]. Meta-analysis of oxidative stress-mediated damage showed a significant increase in TBARS levels in schizophrenia patients compared to healthy controls [19]. Moreover, increased levels of TBARS were reported in plasma/serum of neuroleptic-free patients with schizophrenia compared to normal controls [20]. Antipsychotics treatment was found to increase TBARS levels in plasma [21] and CSF [22–24] in chronic schizophrenic patients. The research on animal models suggests that antioxidant supplementations have beneficial effects and may prolong life of patients [17]. Some observations also suggest that antioxidant supplementations may prolong the human life, whereas other studies demonstrate neutral or even harmful effects [25–28]. In schizophrenia, the antioxidant defence system is altered, and activities of antioxidant enzymes are also changed [3, 9, 29]. Moreover, earlier studies showed that in schizophrenic patients reduced status of plasma total antioxidant capacity was observed [8, 30]. In schizophrenia, oxidative stress manifested by increased lipid peroxidation may be also induced by treatment with antipsychotic drugs [31, 32]. Our earlier results showed that treatment with antipsychotics such as haloperidol and ziprasidone caused an increase in lipid peroxidation measured by TBARS level in plasma [33, 34] whereas the second-generation antipsychotics such as olanzapine and risperidone* in vitro* did not cause plasma lipid peroxidation [30, 35]. Decrease of lipid peroxidation induced by amisulpride, clozapine, or quetiapine was also observed [33, 34, 36]. Lohr et al. [22] in the 1990s found that oxidative stress contributed to the toxicity of haloperidol, which activated a sequence of cellular processes leading even to cell death; the production of ROS was an integral part of this cascade. An experimental study in animals showed elevated level of lipid peroxidation and oxidative neuronal injury caused by haloperidol [37].

It is known that the exogenous plant antioxidants present in human diet, such as resveratrol, quercetin, and other polyphenols, may protect against oxidative stress [38, 39]. Since the direct estimation of reactive oxygen species generation* in vivo* is difficult, a common approach to measure oxidative stress* in vivo* and* in vitro* is to determine lipid peroxidation by means of commonly used assay with thiobarbituric acid (TBA). Therefore the aim of this study was to establish* in vitro* the action of ziprasidone (SGA) on lipid peroxidation in human plasma measured by the level of TBARS and determine the effect of polyphenols derived from berries of* Aronia melanocarpa* (Aronox) on this process. Ziprasidone (5-[2-(4-[1,2-benzisothiazol-3-yl]-1-piperazinyl)ethyl]-6-chloro-1,3-dihydro-2H-indol-2-one) was used* in vitro* at final concentrations corresponding to doses used for treatment of acute episode of schizophrenia [40, 41].

## 2. Materials and Methods

### 2.1. Materials

Ziprasidone (active substance) was obtained from Pfizer Inc. (USA) and Aronox (polyphenols) from Agropharm/Adamed (Poland). All other reagents were of analytical grade and were provided by commercial suppliers. Stock solution of ziprasidone and Aronox were made in 0.001% dimethylsulfoxide (DMSO).

### 2.2. Inclusion Criteria of Volunteers

Blood samples were taken from 30 healthy volunteers (15 males and 15 females) aged between 25 and 27 years (average: 25.9; SD = 1.7 years) without psychiatric, neurological, or somatic disorders and history of head injuries, allergy, and lipid or carbohydrate metabolism disorders, untreated with drugs. Healthy subjects did not use addictive substances and antioxidant supplementation and lived in similar socioeconomic conditions, and their diet was balanced (meat and vegetables). Subjects with significant medical illness were excluded. There were no smokers. The psychiatric examination MINI (Mini-International Neuropsychiatric Interview) [42], neurological and somatic examinations, and total cholesterol, LDL, HDL, triglycerides, and glucose concentrations were performed.

All volunteers included in the study had been informed about aims of the study and methods implemented and they expressed their written informed consent for participation in this study. The protocol was passed by the Committee for Research on Human Subjects of the Medical University of Lodz (number RNN/899/2000).

### 2.3. Isolation and Incubation of Plasma with Ziprasidone and Aronox

Human blood from healthy volunteers was collected into sodium citrate (5 mmol/l at final concentration) and immediately centrifuged (3000 × g, 15 min) to get plasma. Ziprasidone solutions were added to the samples of plasma (0.5 mL) to get the final concentration of 40 ng/mL; 139 ng/mL; and 250 ng/mL. For each experiment the control samples (without the drug) were prepared. The plasma samples with ziprasidone (final concentration 40 ng/mL; 139 ng/mL; 250 ng/mL) and the control samples were incubated for 1 and 24 h at 37°C. To measure the effects of plant polyphenols derived from Aronox, the samples of plasma were preincubated for 5 min at 37°C with Aronox solutions in 0,001% DMSO (final concentrations: 5 ug/mL; 50 ug/mL) and then ziprasidone was added. After 1 h or 24 h incubation (37°C) the levels of TBARS were measured.

### 2.4. Estimation of Thiobarbituric Acid Reactive Substances (TBARS) in Plasma

Samples of plasma after 1 or 24 h incubation with ziprasidone alone and/or Aronox were stopped by cooling the samples in an ice bath. Samples of plasma were transferred to an equal volume of 20% (v/v) cold trichloroacetic acid in 0.6 M HCl and centrifuged at 1200 × g for 15 min. One volume of clear supernatant was mixed with 0.2 volume of 0.12 M thiobarbituric acid in 0.26 M Tris, pH 7.0, and immersed in a boiling water bath for 15 min. The absorbance was measured in the SEMCO spectrophotometer at 535 nm, according to the Rice-Evans method [43] modified by Wachowicz et al. [44]. The TBARS expressed in *μ*mol/l were calculated based on the absorbance value, using the molar extinction coefficient for TBARS (*ε* = 1.56 × 10^5^ M^−1^ × cm^−1^). All estimations were performed twice, including control samples, where spontaneous lipid peroxidation, without the influence of the drug on plasma, was measured.

### 2.5. Statistical Analyses

All the values in this study were expressed as mean ± SEM. In order to eliminate uncertain data, Grubbs' test was performed. The statistical analysis of difference between the control plasma (without drug) and plasma treated with ziprasidone or ziprasidone and* Aronia melanocarpa* (Aronox) was done with a paired Student's *t*-test using StatSoft Inc. “Statistica” v. 6.0.

## 3. Results 

**Figure 1:**
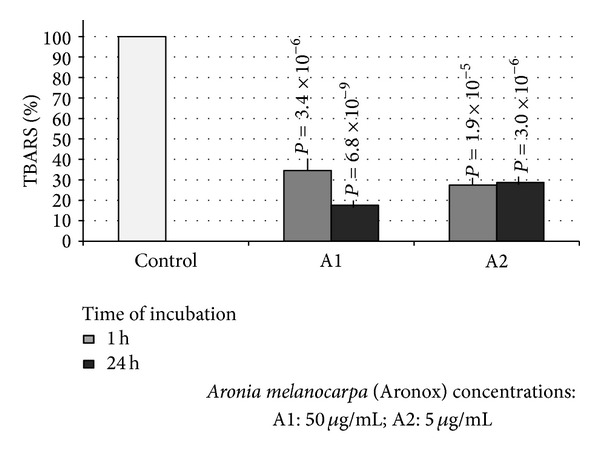
The inhibitory effect of compounds derived from* Aronia melanocarpa *on lipid peroxidation in plasma. Plasma was incubated with 5 *μ*g/mL; 50 *μ*g/mL of Aronox solution for 1 and 24 hours at 37°C. The level of TBARS in control plasma (1 h: 0.712 ± 0.04 *μ*mol/l; 24 h: 1.564 ± 0.06 *μ*mol/l) was expressed as 100%.

**Figure 2:**
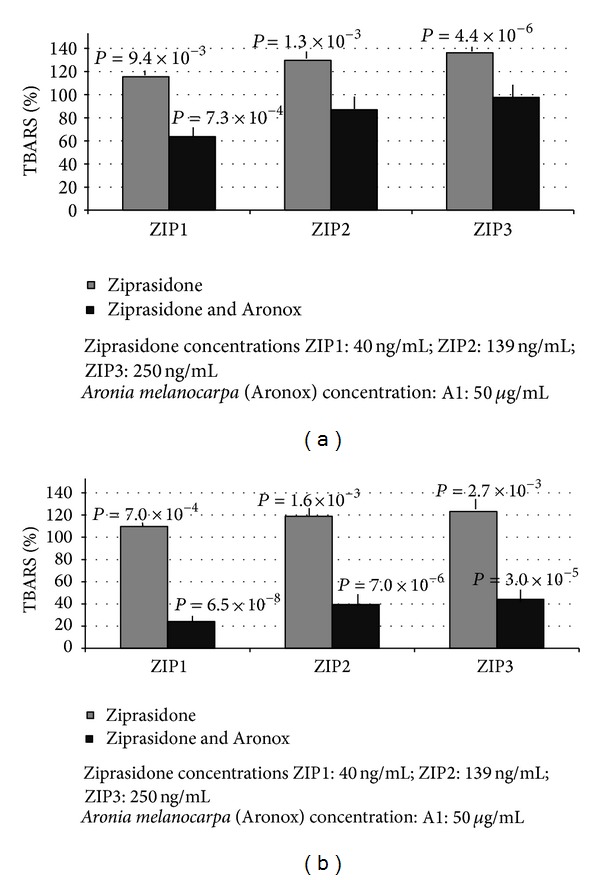
(a) The effects of ziprasidone alone (gray columns) and ziprasidone at the presence of 50 *μ*g/mL Aronox (black columns) on the lipid peroxidation (TBARS) in plasma after 1 h incubation. TBARS level in control plasma (without ziprasidone and Aronox) was expressed as 100%. The level of TBARS in control plasma (1 h: 0.712 ± 0.04 *μ*mol/l). (b) The effects of 24 h incubation of plasma with ziprasidone and ziprasidone with Aronox (50 *μ*g/mL) on the level of lipid peroxidation measured as TBARS. The TBARS level in control plasma (without ziprasidone and Aronox) was expressed as 100%. The level of TBARS in control plasma (24 h: 1.564 ± 0.06 *μ*mol/l).

Our results show that polyphenols from* Aronia melanocarpa* have an inhibitory effect on lipid peroxidation in plasma measured as TBARS level (Figures 1 and 2). We observed the dose-dependent increased level of TBARS after the incubation of plasma for 1 and 24 hours with ziprasidone (final concentrations: 40 ng/mL; 139 ng/mL; and 250 ng/mL) (Figure 2) compared to control samples without drug (1 h: 0.712 ± 0.04 umol/l; 24 h: 1.564 ± 0.06 umol/l, resp.) expressed as 100% (1 h: *P* = 9.4 × 10^−3^; *P* = 1.3 × 10^−3^; *P* = 4.4 × 10^−6^, resp.) (Figure 2(a)) after (24 h: *P* = 7.0 × 10^−4^, *P* = 1.6 × 10^−3^, *P* = 2.7 × 10^−3^, resp.) (Figure 2(b)). The polyphenol extract (final concentrations: 5 ug/mL, 50 ug/mL) caused distinct reduction of TBARS level. The effects are presented in Figure 1. We showed that used concentrations of polyphenols from* Aronia melanocarpa* (Aronox) had inhibitory effects on the increase of TBARS level in plasma induced by ziprasidone. After 1- and 24-hour incubation of plasma with extract from* Aronia melanocarpa* (50 ug/mL), the TBARS level was reduced by about 65% (*P* = 3.4 × 10^−6^) and by 82% (*P* = 6.8 × 10^−9^), respectively (Figure 1). The reduction of TBARS level in plasma treated with ziprasidone (40 ng/mL, 139 ng/mL, and 250 ng/mL) at the presence of Aronox (50 ug/mL) is presented in Figure 2. After 1 h the TBARS levels were reduced by about 51% (*P* = 7.3 × 10^−4^), 42% (*P* > 0.05), and 38% (*P* > 0.05), respectively (Figure 2(a)). After 24 h the TBARS levels was reduced by about 85% (*P* = 6.5 × 10^−8^), 80% (*P* = 7.0 × 10^−6^), and 79% (*P* = 3.0 × 10^−5^), respectively (Figure 2(b)). The reduction of TBARS level in plasma treated with ziprasidone (40 ng/mL, 139 ng/mL, and 250 ng/mL) at the presence of Aronox (5 ug/mL) after 1 h was about 4%, 10%, and 15%, respectively (*P* > 0.05), and after 24 h the TBARS levels were reduced by about 34% (*P* = 6.2 × 10^−3^), 32% (*P* = 1.0 × 10^−3^), and 28% (4.9 × 10^−2^), respectively. The reduction of TBARS level was dependent on the concentration of Aronox (Figure 1).

## 4. Discussion

During oxidative stress in biological systems the most readily accessible reaction partner is the biological membrane, in particular the polyunsaturated fatty acids of the membrane phospholipids. Once the reaction generates a lipid radical, a radical chain reaction occurs, leading to the formation of a variety of lipid peroxidation products which react with thiobarbituric acid and form TBARS. TBARS are very sensitive markers of this process. Oxidative stress-mediated injury is a part of the primary illness for many disorders including schizophrenia and it can be exacerbated by certain neuroleptics [45, 46]. Several studies indicated that the occurrence of movement disorders, including extrapyramidal symptoms in patients with psychotic disorders treated by classic neuroleptics, was associated with their oxidative stress [32]. In the cerebrospinal fluid of patients treated with antipsychotics, the concentration of TBARS was found to be increased [23], especially in patients with drug-induced movement disorders [22]. In schizophrenic patients with symptoms of tardive dyskinesia the lipid peroxidation in plasma was found to be increased [21]. Our earlier studies by using various specific biomarkers of oxidative stress revealed that in peripheral blood platelets or plasma from schizophrenic patients (in an acute period of psychosis) increased lipid peroxidation [3, 30] and oxidative/nitrative modifications of proteins [47, 48] occurred. According to other authors the antipsychotic treatment (especially FGAs) exhibits prooxidative effects [22, 32], whereas SGAs may exhibit antioxidative effects [32, 35]. Our earlier results indicate that SGAs, including clozapine, quetiapine, and olanzapine (at the concentrations proposed for therapeutic use in schizophrenic patients [49], did not induce plasma lipid peroxidation* in vitro* [33, 34]. Risperidone, even at very high concentrations, did not induce oxidative stress in plasma or in blood platelets [35].

The results presented in this study confirmed our earlier observation that ziprasidone induces lipid peroxidation in plasma [34]. Epidemiological and clinical study evidence suggests that a diet rich in fruits and vegetables decreases the risk of premature mortality from major clinical conditions, including heart disease and cancer. Recently, much attention has focused on possible role of diet in the prevention of chronic diseases [25, 50, 51]. In the present* in vitro* study, we assessed the effects of polyphenols present in* Aronia* extract on TBARS level in plasma treated with ziprasidone. The extract from* Aronia melanocarpa (Rosaceae)* containing anthocyanidins, phenolic acids, and quercetine glycosides may play an important protective role against lipid peroxidation. Phenolic compounds found naturally in fruits, nuts, flowers, seeds, and bark of different plants are an integral part of human diet.

They exhibit a wide range of biological effects, including antiplatelet, anti-inflammatory, anticancer, antimutagenic, and antifungal properties [51–53]. They are potent antioxidants and scavengers of reactive oxygen species and metal chelators. Some polyphenols like quercetin reverse haloperidol-induced catalepsy during schizophrenia or other affective disorders [54]. Here, we have shown for the first time that polyphenols present in* Aronia melanocarpa* extract* in vitro* distinctly reduced lipid peroxidation of plasma treated with ziprasidone. We conclude that natural products present in extract of* Aronia melanocarpa* may have some promising effects* in vivo* because they are strong antioxidants in the tested models* in vitro*. It seems that polyphenols from* Aronia melanocarpa* can be also useful as protecting factors against antipsychotic drugs used during schizophrenia. The life style of schizophrenic patients with low consumption of fruits and vegetables and treatment with prooxidative drugs leads to increased oxidative stress. Therefore the supplementation with antioxidants such as Aronox rich in polyphenols with antioxidant properties may have beneficial effects on the improved outcome of illness.

Plasma total antioxidant status (TAS) was significantly lower in schizophrenia subjects than in normal controls. Recently, reduced levels of plasma TAS have been shown in first-episode drug-naive patients with schizophrenia [55]. Individual plasma antioxidants, albumin, bilirubin [8, 56], and uric acid [8] were also found to be lower in schizophrenia subjects. Moreover, decreases of total and reduced GSH levels in plasma together with altered antioxidant enzyme activities have been reported in drug-naive first-episode patients with schizophrenia when compared with healthy control subjects [57].

In schizophrenia subjects, the mean values of serum MDA (malonyldialdehyde) levels were found significantly higher than that in the healthy individuals [10, 58]. Similar results were also observed in the blood of chronic schizophrenic patients compared to normal controls [59, 60]. Moreover, elevated serum TBARS were also found in different phases of bipolar disorder and in schizophrenia [61]. It seems that oxidative stress due to higher lipid peroxidation might play a critical role in pathogenesis of schizophrenia at very early course of illness.

Although antipsychotic drugs are the first choice of treatment for schizophrenia, their long-term treatment is known to produce adverse effects and may be responsible for increased lipid peroxidation and oxidative damage. Oxidative stress induced by antipsychotics may be counteracted by antioxidant enzymes and exogenous antioxidants. Therefore, it is essential to develop adjunctive or alternative treatment strategy to augment antipsychotic actions and improve the quality of life for schizophrenia patients. Some clinical, preclinical, and epidemiological studies suggest that many of antioxidant compounds present in fruits and vegetables, especially polyphenols, possess neuroprotective and anti-inflammatory activities, to greater or lesser extent, and could be considered as important adjunctive therapy for schizophrenia [36, 54]. We suggest that Aronox containing few important polyphenolic antioxidants might be used for the therapeutic purpose in this disorder; however more studies are required. Carefully monitored clinical trials in the future will provide more information regarding the effects of extract from* Aronia melanocarpa* in patients treated with antipsychotics.
